# State‐Guided TMS‐EEG for N100 Enhancement Study Based on Whole‐Brain EEG Microstates

**DOI:** 10.1002/cns.70975

**Published:** 2026-06-15

**Authors:** Jiale Lan, Yong Wang, Xiaoli Li, He Chen

**Affiliations:** ^1^ School of Automation Science and Engineering, South China University of Technology Guangzhou China; ^2^ Department of Rehabilitation Medicine Zhujiang Hospital, Southern Medical University Guangzhou China; ^3^ State Key Laboratory of Cognitive Neuroscience and Learning Beijing Normal University Beijing China

**Keywords:** EEG microstates, state‐guided stimulation, TMS evoked N100, TMS–EEG

## Abstract

**Objective:**

Transcranial magnetic stimulation‐electroencephalography (TMS‐EEG) enables the non‐invasive assessment of cortical excitability and inhibition. However, the N100 component‐a key marker of cortical inhibition‐often exhibits poor trial‐to‐trial stability, limiting its utility as a reliable readout. The aim of this study is to propose a whole‐brain, state‐guided TMS‐EEG protocol for enhancing TMS‐evoked N100.

**Methods:**

TMS‐EEG data were acquired from 19 healthy adults. After standard preprocessing, single trials were sorted into five datasets according to the microstate present at the time of stimulation. For each microstate‐specific dataset, global and local mean field amplitudes (GMFA/LMFA) and TMS‐evoked potentials (TEPs) were computed to compare N100 characteristics across microstates.

**Results:**

TMS delivered during the S4 microstate produced the numerically largest N100 values in the GMFA and LMFA. However, these differences did not reach statistical significance and should therefore be interpreted as exploratory descriptive findings. The clearest statistically significant microstate effect was observed for the TEPs. The N100 component of S4 group (−2.38 μV; |N100| = 2.38 ± 0.33 μV) was significantly higher than S2 group (*p*_adj_ = 0.030), and S1 also elicited a larger response than S2 (*p*_adj_ = 0.012). This pattern was most clearly retained after moderate spatial downsampling to 32 channels, whereas the S4‐related evidence became weaker and was no longer statistically retained at lower electrode densities. At 64 channels, S4 showed a significantly larger absolute N100 amplitude than S2 (*p*_adj_ = 0.030), and S1 was also significantly larger than S2 (*p*_adj_ = 0.013). At 32 channels, both contrasts remained significant, with S4 exceeding S2 (*p*_adj_ = 0.034) and S1 exceeding S2 (*p*_adj_ = 0.013). At 21 channels, only the S1 versus S2 contrast survived correction (*p*_adj_ = 0.025), whereas no S4‐related pairwise contrast survived. At 9 channels, no pairwise comparison survived correction.

**Conclusion:**

Based on offline analysis, we established and validated a whole‐brain, state‐guided TMS‐EEG framework for stabilizing and amplifying the N100 component. Stimulation delivered during the S4 microstate produced a larger local TEP N100 response, whereas GMFA and LMFA were analyzed as exploratory descriptive outcomes and showed S4‐related numerical patterns. Moreover, the S4‐related N100 enhancement remained statistically supported after downsampling from 64 to 32 channels, but was not retained in the 21‐ or 9‐channel montages. These findings indicated that microstate‐based state monitoring may offer a viable framework for future state‐guided neuromodulation in both research and clinical applications.

## Graphical abstract

This study proposes a whole‐brain, state‐guided TMS‐EEG framework to enhance the TMS‐evoked N100, with the most evident effect observed for peak N100 TEP amplitude and detectable effects in GMFA and LMFA. This pattern was retained in 64‐, 32‐, and 21‐channel montages, while a similar trend was preserved in the 9‐channel configuration. This framework may support more robust and interpretable N100 readouts in TMS‐EEG research.

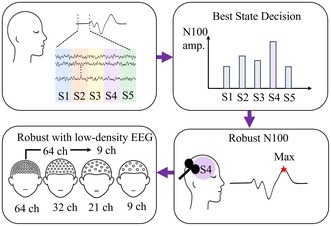

## 1 Introduction

Transcranial magnetic stimulation (TMS) is a non‐invasive brain stimulation technique that can be used to activate cortical neurons [1, 2]. Combined with electroencephalography, TMS‐EEG enables millisecond‐scale characterization of cortical and large‐scale network dynamics [3, 4, 5], and has expanded from basic research to emerging clinical applications (e.g., diagnostic and therapeutic monitoring) [6]. In a typical TMS‐EEG paradigm, brief magnetic pulses are delivered to a targeted cortical region, and the induced neural activity elicits a sequence of TMS‐evoked potentials (TEPs) recorded at the scalp [7]. When stimulating the motor cortex or frontal cortex, TEP components primarily comprise the P30 (approximately 30 ms), N45 (40–50 ms), P60 (60–70 ms), N100 (80–120 ms), P180 (160–200 ms), and N280 (250–350 ms) [3, 4, 8, 9, 10, 11]. These components index excitation‐inhibition processes and network‐level interactions, and are increasingly explored as biomarkers for neuropsychiatric disorders [6, 12, 13, 14, 15].

However, TEP stability remains a major concern [16, 17]. TEP signals exhibit substantial variability, and the amplitudes of individual components are influenced by inter‐individual factors such as age‐related brain maturation [5, 18, 19], and are susceptible to secondary sensory‐related artifacts [15, 20]. Therefore, TEPs also typically have low signal‐to‐noise ratios, which necessitates many repetitions to obtain stable waveforms [17]. Collectively, these factors limit the reliability of TMS‐EEG measurements and hinder their broader implementation in clinical practice. Multiple studies have shown that transient brain state characteristics such as the phase and power of neural oscillations strongly modulate the amplitude and morphology of TMS‐evoked responses [21, 22]. These findings suggest that state‐targeted stimulation may improve TEP stability by delivering TMS in advantageous intrinsic brain states.

In recent years, extensive research has demonstrated that modulating local brain state characteristics (such as the phase or power of neural oscillations) can alter the strength of evoked neurophysiological responses [22, 23, 24]. Consistent with state‐dependent excitability frameworks, converging evidence supports a causal link between ongoing oscillatory states and immediate TMS effects [25, 26, 27, 28]. However, these approaches focus on local properties, whereas spontaneous activity is organized at the whole‐brain level: the resting brain transitions among distinct global functional modes [29]. Therefore, it is important to test how global brain‐state patterns shape TMS‐evoked responses. Accumulating evidence indicates that TMS‐EEG can be used to assess effective connectivity within large‐scale networks [30]. Notably, TEP components emerging approximately 100 ms after stimulation, including the canonical N100 time window, primarily reflect whole‐brain network responses rather than local potential fluctuations at the stimulation site [31]. Given that late TEP components (such as the N100) depend on coordinated whole‐brain network activity, modulation strategies based on whole‐brain states are more closely aligned with the underlying physiology than approaches that rely solely on local oscillatory phase or power. Scalp topography‐based EEG microstates provide a means to characterize such whole‐brain states by integrating spatial information across electrodes and indexing global functional configurations.

EEG microstates, first proposed by Lehmann et al. in 1987 [32], are quasi‐stable topographic states derived from EEG signals, typically lasting 80 to 120 ms [33]. By integrating spatial information across the entire electrode array, microstate analysis indexes global functional configurations, providing a robust framework for characterizing whole‐brain functional states [34]. In TMS‐EEG studies, microstate analysis has mainly been used to relate pre‐stimulation brain states to variability in TMS‐evoked responses and to characterize post‐TMS whole‐brain dynamics beyond conventional TEP analysis [35, 36]. Existing work further shows that specific microstates modulate responses to external stimuli and that stimulation delivered during particular microstates can enhance response magnitude [35, 37], thereby providing convergent evidence that global brain states can effectively regulate neural responsiveness.

Although TMS‐EEG elicits multiple TEP components (P30‐N45‐P60‐N100‐P180‐N280) in healthy adults, we focused on the N100. This focus does not overlook the functional significance of earlier latencies. Recent studies suggest that TMS‐evoked activity can reflect distributed network recruitment at earlier latencies [38], and that stimulation parameters can shape cortico‐cortical activation and network‐level dynamics well before 100 ms [36]. This view is further supported by microstate and functional connectivity studies of post‐TMS activity [39]. Nevertheless, the N100 remains a useful focus because it is widely considered an index of cortical inhibition, sensitive to GABA_*B*_‐mediated inhibition and the local glutamate/GABA balance, and it exhibits systematic age‐related changes [18, 40, 41]. In addition, N100‐related measures have been shown to be sensitive to plasticity‐related stimulation protocols and other neuromodulatory interventions that alter cortical inhibition [42, 43], supporting their use as candidate biomarkers of inhibitory and plasticity‐related cortical functioning. It is also increasingly viewed as a biomarker of GABA_*B*_‐mediated inhibition and cortical dysfunction in depression, schizophrenia, and cognitive impairment [44, 45]. Clinically, prefrontal N100 amplitude has been associated with subsequent reductions in suicidal ideation in depression [40], and reduced N100 has been reported in children with ADHD [46]. Consequently, these converging findings underscore the functional significance of the N100 and suggest that identifying brain states associated with larger N100 responses may provide a foundation for future translational investigations of inhibitory processing.

However, existing state‐dependent strategies to enhance N100 primarily rely on local features (phase or power) and require stable rhythmic activity in the target region [47], which can lead to substantial variability in induction effects [48]. This problem is particularly pronounced in non‐motor cortical regions, such as the dorsolateral prefrontal cortex (DLPFC), where the stability and amplitude of α and θ band activity are markedly lower than the μ or β rhythms typically observed in the primary motor cortex (M1) [49]. Consequently, both the accuracy of online phase estimation and the probability of successfully hitting the desired state are markedly reduced.

To address these limitations, we propose an EEG microstate‐based approach to stabilize and enhance the N100 component. Unlike strategies that rely on local oscillatory phase or power within a target region, EEG microstates characterize quasi‐stable, large‐scale patterns of whole‐brain activity. On this basis, we developed a whole‐brain state‐guided N100 enhancement protocol: the ongoing global brain state at stimulation onset was identified using conventional microstate segmentation methods, and trials were subsequently grouped according to specific microstate patterns to examine whether stimulation delivered during a specific microstate could achieve larger N100 amplitude. In parallel, to reduce the reliance of current state‐guided TEP paradigms on high‐density EEG configurations, we systematically evaluated influences of electrode density on the detectability and spatial specificity of the N100.

## 2 Methods

### 2.1 Materials

#### 2.1.1 Equipment

This study used a TMS‐compatible BrainAmp amplifier (Brain Products, Germany) with a 64‐channel TMS‐compatible Ag/AgCl electrode cap (EASYCAP GmbH, Germany). Electrodes followed the international 10–20 layout [50]. FCz was used as the online reference. The sampling rate was 2500 Hz. Electrode impedances were kept < 5 kΩ. TMS was delivered using a Magstim *R* ^2^ stimulator with a 70‐mm figure‐of‐eight coil over F3 (DLPFC), held tangential to the scalp at ~45° to the midline to induce a posterior‐to‐anterior current. Auditory/bone‐conducted artifacts were minimized using continuous white‐noise masking (insert earphones) and a thin foam spacer. Pulses were delivered every 2.1–2.5 s at 90% resting motor threshold (RMT; ≥ 50 μV MEPs in ≥ 5/10 trials from the relaxed first dorsal interosseous muscle). Sessions lasted ~45–60 min, and no participants took sedative medications.

#### 2.1.2 Participants and Protocol

Nineteen healthy adults (mean age = 53.16 ± 7.68 years old; eight men, eleven women), all right‐handed, technical secondary school to university level, and without a history of neurological disorder, took part in the study. The study was approved by the Ethics Committee of the State Key Laboratory for Cognitive Neuroscience and Learning at Beijing Normal University (Approval number: CNL_A_0010_010). All participants provided written informed consent, and this study was conducted in accordance with the Declaration of Helsinki.

### 2.2 Pre‐Processing

**FIGURE 1:**
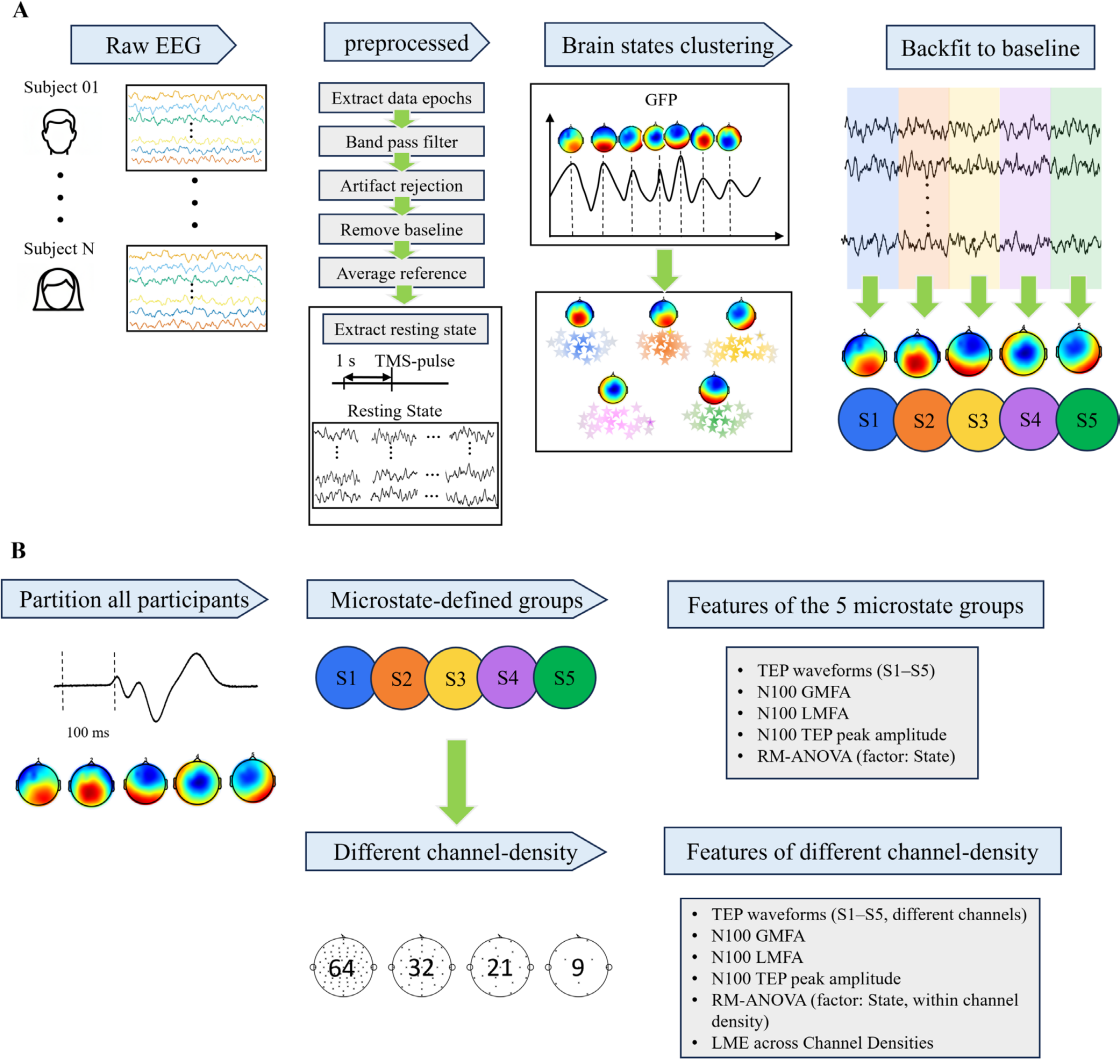
Analysis pipeline. (A) Overview of the process for extracting microstate. (B) Quantitative feature analysis for each microstate.

In this study, we preprocessed the TMS‐EEG data by the TMS‐EEG signal analyzer software (TESA) [51] in MATLAB (R2020a, Mathworks, USA). Figure 1A summarizes the preprocessing workflow and the extraction of 1‐s pre‐stimulation resting‐state segments. Briefly, the preprocessing consisted of the following steps: (1) imported raw data and set electrode locations; (2) detected the bad channels and removed them using the *clean_channels* function (correlation threshold = 0.7); (3) epoched data from −1000 to 1000 ms relative to each TMS pulse, baseline‐corrected using −500 to 0 ms, removed the TMS pulse artifact and early high‐amplitude muscle‐related peaks (−10 to 10 ms), and interpolated the missing segment using a cubic interpolation function of TESA; (4) visually inspected epochs, rejected poor‐quality epochs (deleted epochs: 8.53 ± 7.11, retained epochs: 141.47 ± 9.38); (5) EEG data were down‐sampled to 1000 Hz and filtered using zero‐phase Butterworth band‐pass filter (1–40 Hz) to eliminate line noise and improve subsequent preprocessing steps; (6) Two rounds of ICA by FastICA were performed: the first to remove components associated with TMS‐evoked muscle, electrical, and movement artifacts, and the second to extract and remove components associated with blinks, eye movements, sustained muscle activity, and electrode noise. Independent components (ICs) were identified as artifactual based on the spatial topography of their weights, trial‐by‐trial activity, mean evoked activity, spectral transformation, and component time series, and were then rejected (20.79 ± 5.97); and (7) interpolated bad channels using a spherical spline method and re‐referenced to the common average.

### 2.3 Microstate Template Generation and Dataset Segmentation

Microstate analysis was performed in MATLAB using the open‐source Microstate EEGLAB toolbox (v1.0; Figure 1). For each participant, global field power (GFP) was computed from concatenated 1‐s pre‐stimulus EEG segments recorded immediately before each TMS pulse, and was defined as the spatial standard deviation across electrodes at each time point [52]. Scalp maps at GFP peaks were clustered using modified k‐means to derive candidate microstate classes (*k* = 3–8) [53, 54], with a maximum of 1000 iterations. For each participant, 1000 GFP peaks (minimum inter‐peak interval: 10 ms) entered segmentation. Based on repeated runs, five microstate maps (S1–S5) were retained and back‐fitted to the 1‐s pre‐TMS EEG to obtain a label time series. Given typical microstate durations of 80–120 ms [33], the stimulation state was defined from the 100 ms immediately preceding TMS onset. A “random” condition was defined from the original data without microstate partitioning. Specifically, this condition comprises the pooled all‐trial dataset analyzed without microstate classification, serving as a non‐partitioned reference rather than an independent control. Data were then split into five microstate‐specific .set files per participant for subsequent analyses.

### 2.4 TEP

**FIGURE 2:**
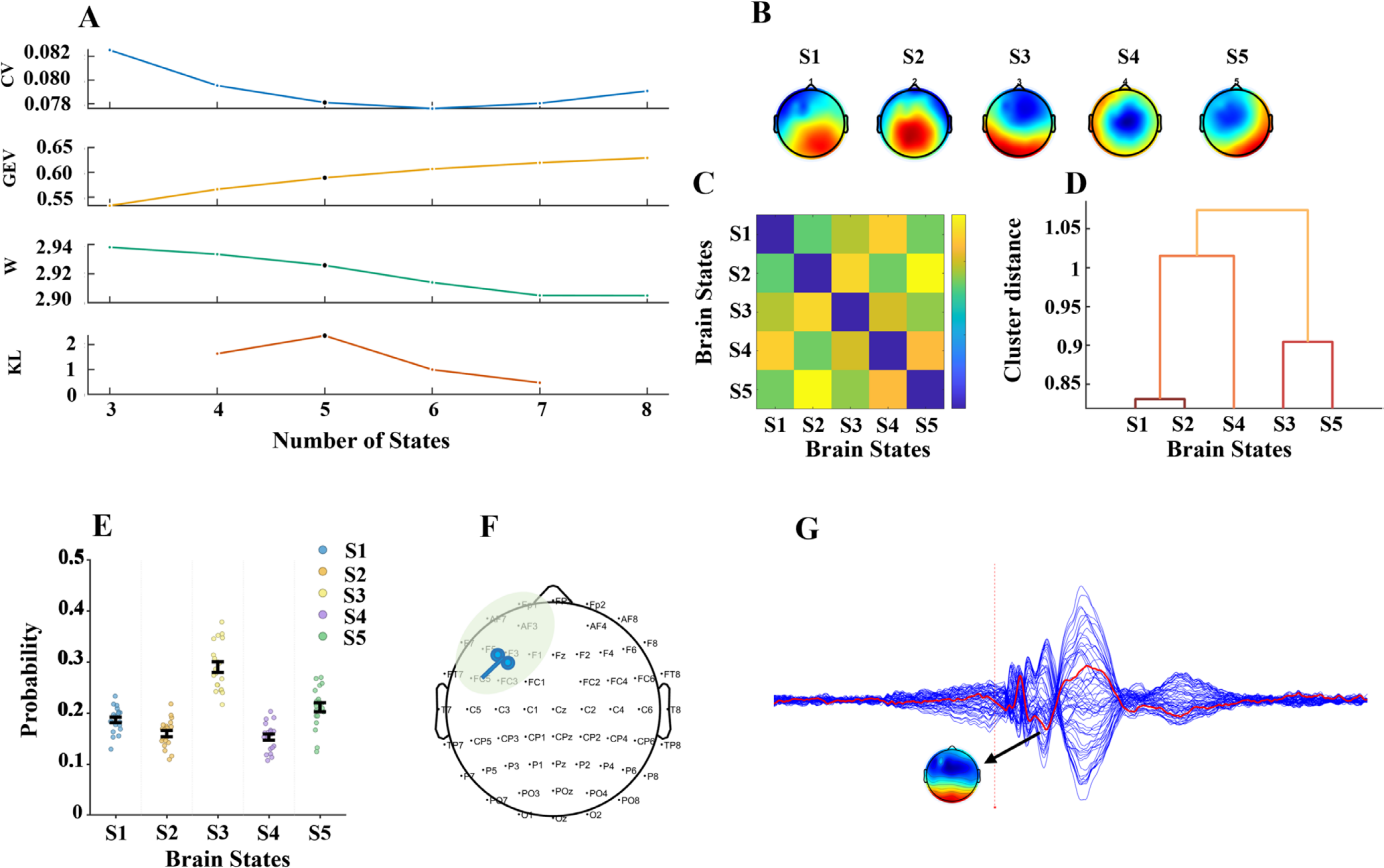
EEG microstate selection and characterization. (A) Model‐selection curves for candidate numbers of microstates (*k* = 3–8): cross‐validation error (CV), global explained variance (GEV), within cluster variance (W) and Krzanowski‐Lai index (KL). (B) Group‐level scalp topographies of the five microstate templates (S1–S5). (C) Spatial similarity matrix between microstate templates. (D) Hierarchical clustering dendrogram of S1–S5 based on spatial distance. (E) Occurrence probability of each microstate within the stimulus‐locked time window; dots show single‐subject values and black bars indicate group mean ± SEM. (F) Stimulation target for TMS and ROI for TEP analysis (highlighted in green shading). (G) TEPs over left DLPFC, average TEP waveform of ROI is indicated by red line, and the topographical distributions of surface EEG for the N100 component.

TMS over the left dorsolateral prefrontal cortex (DLPFC) elicited canonical TEP components; here we focused on the N100, a negative deflection with a midfrontal topography (Figure 2G), consistent with prior TMS‐EEG work [35]. N100 was quantified within 75–150 ms post‐stimulation. For ROI‐based analyses, signals from nine channels surrounding the target (Fp1, AF7, AF3, F5, F3, F1, FC5, FC3, FC1; Figure 2F) were averaged to obtain an ROI waveform. We computed two summary measures: global mean field amplitude (GMFA), the spatial standard deviation across all electrodes at each time point, and local mean field amplitude (LMFA), defined analogously but restricted to the ROI channels.

### 2.5 Electrode Montage Configurations

To evaluate N100 across electrode densities (64, 32, 21, and 9 channels), we simulated lower‐density montages by selecting the corresponding electrode subsets from the 64‐channel array. The frontal ROI was defined as Fp1/F3/AF3/FC1/FC5 for the 32‐channel montage, Fp1/F3 for the 21‐channel montage, and F3 for the 9‐channel montage. Electrode selection followed the international 10–20 system to approximate standard cap layouts. All analyses used the same processing pipeline, temporal windows, and definitions as in the 64‐channel analysis; only the input channel set differed across density conditions.

### 2.6 Pre‐ **TMS** Scalp Topography Reconstruction and Global Amplitude Index

Based on the baseline period preceding each TMS pulse, baseline samples assigned to each microstate were pooled across trials to derive a state‐specific scalp potential map. For each subject and state, we averaged the pooled samples over time for each electrode to obtain a vector of electrode‐wise mean voltages. To quantify the global amplitude, we summed the absolute electrode‐wise means across channels, avoiding polarity cancellation and yielding a single index of overall field strength for that state. In the Results, maps are shown as 2D topographies and their global amplitudes are compared across states.

### 2.7 Pre‐TMS Scalp Temporal Complexity Analysis

To quantify microstate‐specific temporal complexity, we computed single‐channel Lempel–Ziv complexity (LZs) and summarized it as a global index (LZs_global_). For each microstate, samples labeled within the pre‐TMS window (−100 to 0 ms) were pooled across trials and concatenated to form a microstate‐specific time series per channel, following the Lempel–Ziv EEG diversity framework of Schartner et al. [55] adapted to the pre‐stimulation microstate window. Instantaneous amplitudes were obtained via Hilbert transform and binarized using channel‐specific mean Hilbert amplitude over the baseline period as the threshold. LZs was then normalized by sequence length *n* [56]. Finally, LZs_global_ was computed as the mean LZs across all channels for each microstate, where higher values indicate greater temporal diversity (i.e., lower compressibility) of pre‐TMS scalp activity.

### 2.8 Topographic Dissimilarity Between Microstate Templates

Topographic dissimilarity between microstate templates was quantified using correlation‐based global map dissimilarity (GMD) [57]. Group‐level maps for S1–S5 were represented as scalp potential vectors $v_{k}$. Each $v_{k}$ was mean‐centered and L2‐normalized, and pairwise similarity was computed as the absolute spatial Pearson correlation $|r_{\mathrm{kl}}|$. GMD was defined as

(1) $${\mathrm{GMD}}_{k\ell }=\sqrt{2\left(1-|r_{k\ell }|\right)}$$

By construction, ${\mathrm{GMD}}_{k\ell }=0$ for identical or polarity‐reversed maps, yielding a polarity‐invariant index of scalp‐map dissimilarity [58]. The resulting 5 × 5 GMD matrix was visualized as a heat map, and agglomerative hierarchical clustering (average linkage) was applied to obtain a dendrogram summarizing template similarity [59].

### 2.9 Covariate‐Adjusted Analysis

To examine whether the microstate‐related N100 effect remained after controlling for baseline field strength, a covariate‐adjusted linear mixed‐effects model was fitted [60]. N100 amplitude was entered as the dependent variable, with State as a categorical fixed effect, mean‐centered pre‐TMS global field amplitude as a continuous covariate, and Subject as a random intercept. A State × pre‐TMS global field amplitude interaction was tested as a robustness analysis. Adjusted marginal means and Bonferroni‐corrected S4‐focused post hoc contrasts were estimated from this model.

### 2.10 Statistical Analysis

- Analyses were performed in SPSS Statistics (IBM; v20.0). Microstate effects on TMS‐EEG measures were tested using repeated‐measures ANOVA with microstate (S1–S5) as the within‐subject factor. Greenhouse–Geisser corrections were applied when sphericity was violated (Mauchly's test), and Bonferroni‐adjusted post hoc tests used a corrected threshold of *p* = 0.05.

To test whether N100 amplitude differed across ROIs while controlling for microstate condition, we fitted a linear mixed‐effects model [61, 62] with ROI and State as fixed effects and a subject‐specific random intercept (SID). Main effects were evaluated using Type III ANOVA with Satterthwaite degrees of freedom. State‐adjusted estimated marginal means (±95% CI) were computed on an equally weighted State reference grid, followed by ROI‐wise contrasts; multiplicity was controlled using Bonferroni and FDR. Model assumptions were assessed using residual normality and homoscedasticity diagnostics.

## 3 Results

### 3.1 Detection of EEG Brain States

To determine the optimal number of microstates *k*, we fitted group‐level templates for *k* = 3–8 (Figure 2A) and evaluated global explained variance (GEV), cross‐validation error (CV), the Krzanowski–Lai index (KL), and within‐cluster variance (W). GEV and W showed diminishing returns with increasing *k*, CV reached a minimum, and KL exhibited local peaks. We jointly considered the GEV/W inflection points, the CV minimum, and KL peaks; when criteria disagreed, we selected a conservative solution and set *k* = 5. Five microstate templates (S1–S5) were obtained (Figure 2B). GMD and hierarchical clustering (Figure 2C,D) revealed two topographic modules: {S1, S2, S4} and {S3, S5}. S1 and S2 were most similar, S3 and S5 clustered together, and S4 joined {S1, S2} at a higher linkage distance, indicating an intermediate but least‐similar position within its module. Pre‐stimulation occurrence probabilities (Figure 2E) tended to be slightly higher for S3 and S5 than for S1, S2, and S4 (mean ± SEM).

### 3.2 TEPs

**FIGURE 3:**
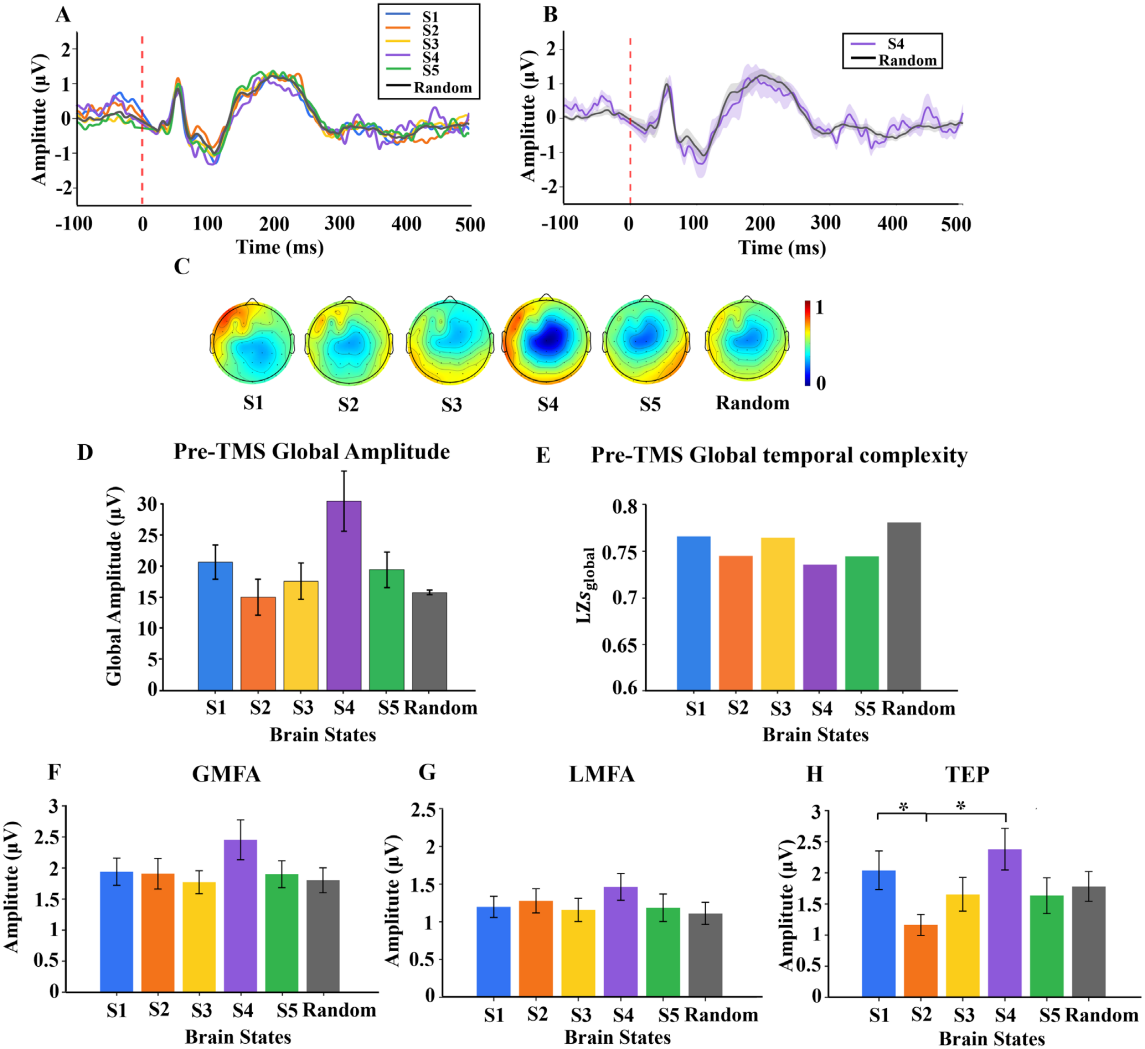
TMS‐evoked potentials across microstate‐defined stimulation conditions. (A) Grand‐average TEP waveforms for the five microstate stimulation conditions (S1‐S5) and the random stimulation condition. (B) Comparison of TEP waveforms between the S4 stimulation condition and the random stimulation condition. (C) Group‐averaged pre‐TMS scalp topographies for the five microstate‐specific conditions and the random condition. (D) Pre‐TMS Global Field Amplitude across the five microstate and random conditions. (E) Scalp topographic complexity index of the pre‐TMS maps for the five microstate stimulation conditions and the random stimulation condition. (F) N100 global mean field amplitude (GMFA) for the five microstate stimulation conditions; error bars, SEM. (G) N100 local mean field amplitude (LMFA) for the five microstate stimulation conditions; error bars, SEM. (H) N100 TEP peak amplitudes for the five microstate stimulation conditions; *p < 0.05, error bars, SEM.

Figure 3A–E summarizes the TEP waveforms and aggregated metrics. A left frontal ROI was defined using electrodes near the stimulation target (Fp1, AF7, AF3, F5, F3, F1, FC5, FC3, FC1; Figure 2F), and ROI TEP waveforms were obtained by averaging across these channels. Figure 3A shows ROI‐averaged TEPs for the five microstates (S1–S5) and the random condition (no microstate classification). A clear N100 component (~75–150 ms) was present in all conditions, and the largest N100 peak was observed in S4. Figure 3B further compares the mean TEP (mean ± SEM) between S4 and the random condition, showing higher N100 amplitude in S4. Pre‐TMS scalp potential maps (Figure 3C) revealed that S4 exhibited a markedly distinct configuration, characterized by a large fronto‐central negative field surrounded by a ring of positive potential, with greater field strength and spatial extent than the other microstates. Consistently, the global amplitude metric (*A*_*s*_) (Figure 3D) was highest in S4, exceeding S1–S3, S5, and the random condition, indicating the greatest overall potential strength immediately before the TMS pulse. As illustrated in Figure 3E, global temporal complexity (LZs_global_) was highest in the random condition and lowest in S4.

Summary statistics are provided in Figure 3F–H. For N100 GMFA, no statistically significant differences were found between microstates; nevertheless, mean values remained numerically highest in S4 (2.43 ± 0.31 μV), followed by S1 (1.93 ± 0.22 μV), S2 (1.90 ± 0.24 μV), S5 (1.89 ± 0.21 μV), the random condition (1.80 ± 0.20 μV), and S3 (1.77 ± 0.18 μV). ROI LMFA results (Figure 3G) showed a similar pattern within the left frontal ROI, with the largest N100 in S4 (1.46 ± 0.18 μV, *n* = 19) and lower mean amplitudes for S2 (1.28 ± 0.16 μV), S1 (1.20 ± 0.14 μV), S5 (1.19 ± 0.18 μV), S3 (1.16 ± 0.15 μV), and the random condition (1.11 ± 0.15 μV); additional ROI‐wise N100 analyses are reported in the Supporting Information Results (Figure S1). For N100 amplitudes summarized in Figure 3H, microstate exerted a significant main effect (*F* = 5.07, *p* = 0.0108, partial *η* ^2^ = 0.22). For descriptive purposes, N100 amplitudes were converted to absolute values and ordered by mean magnitude: S4 (2.38 ± 0.33 μV) > S1 (2.04 ± 0.31 μV) > random (1.78 ± 0.24 μV) > S3 (1.65 ± 0.27 μV) > S5 (1.63 ± 0.29 μV) > S2 (1.17 ± 0.17 μV; mean ± SEM, *n* = 19). Bonferroni‐corrected post hoc tests indicated significant differences between S1 and S2 (Δ = 0.87 μV, *p*_adj_ = 0.012) and between S4 and S2 (Δ = 1.21 μV, *p*_adj_ = 0.030).

As a supplementary robustness analysis, a covariate‐adjusted model was conducted to examine whether the S4‐related N100 pattern remained after accounting for pre‐stimulus global field amplitude. In this model, the main effect of State remained significant (*F*(4, 89) = 5.63, *p* < 0.001; likelihood‐ratio test *χ* ^2^(4) = 19.70, *p* < 0.001), whereas pre‐TMS global field amplitude was not a significant predictor of N100 amplitude (*χ* ^2^(1) = 0.90, *p* = 0.342). The State × pre‐TMS global amplitude interaction was also not significant (*χ* ^2^(4) = 5.71, *p* = 0.222). Adjusted marginal means showed that S4 retained the largest adjusted N100 response. Bonferroni‐corrected S4‐focused contrasts further showed that S4 had significantly larger N100 responses than S2, S3, and S5, whereas the difference between S4 and S1 was not significant. Full model results and adjusted means are available in Table S1.

### 3.3 Electrode Density Effects

**FIGURE 4:**
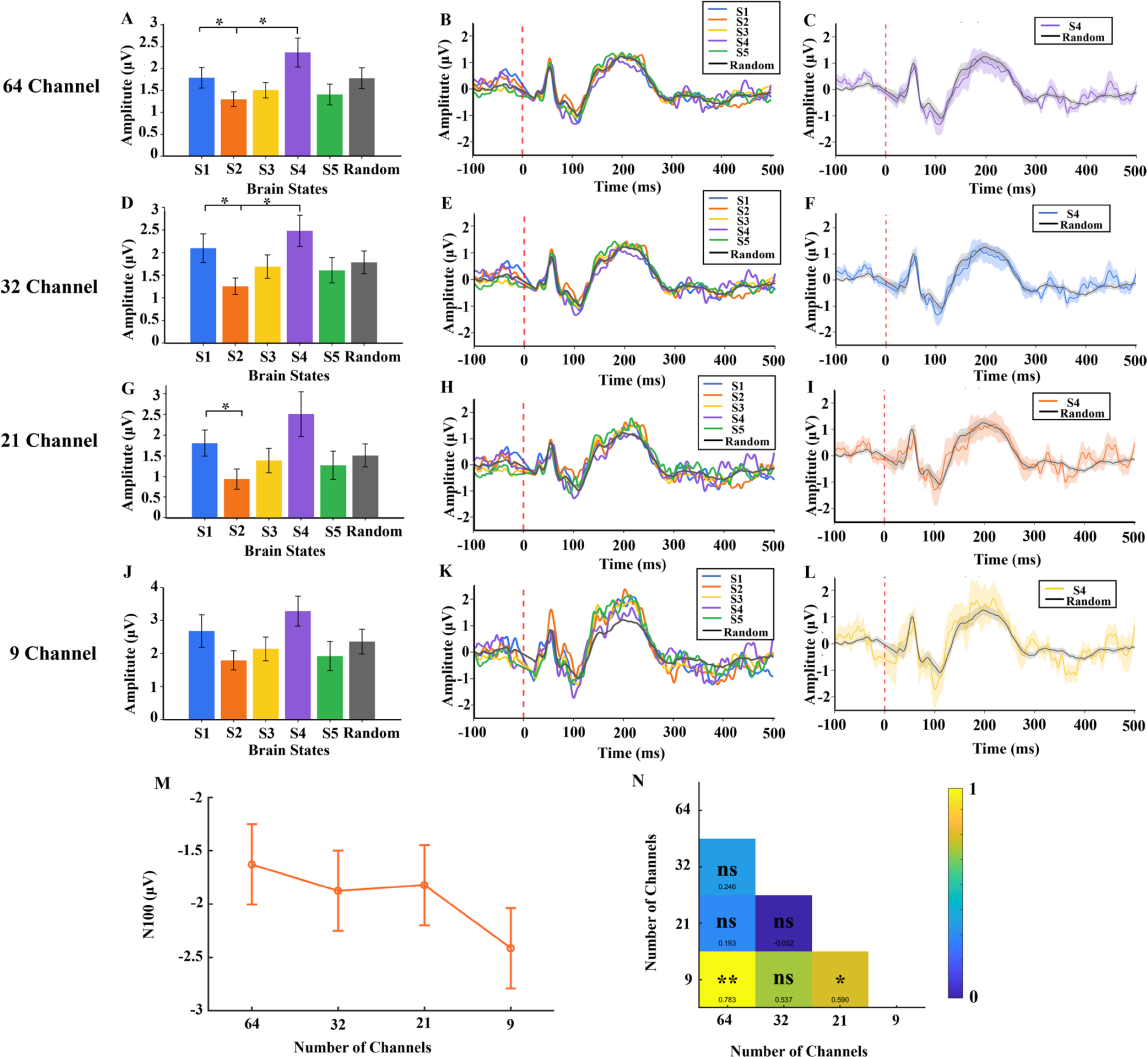
Results of state‐ guided dependent N100 on EEG channel density. (A–C) Results for the 64‐channel montage. (A) N100 peak amplitudes for the five microstate‐locked conditions (S1–S5) and the random timing condition; bars indicate the mean, error bars indicate SEM; *p < 0.05. (B) Grand‐average TEP waveforms for S1–S5 and the random condition (0 ms = TMS pulse). (C) Grand‐average TEP waveforms for the S4 and random conditions with ±SEM shading. (D–F) Results for the 32‐channel montage, arranged as in (A–C). (G–I) Results for the 21‐channel montage, arranged as in (A–C). (J–L) Results for the 9‐channel montage, arranged as in (A–C). (M) Estimated marginal means of N100 amplitude across electrode‐density ROIs (9‐, 21‐, 32‐, and 64‐channel montages; EMM ± 95% CI). (N) Pairwise differences in N100 amplitude between electrode‐density ROIs (Bonferroni‐corrected significance) (*p < 0.05, ns = non‐significant).

To enhance the clinical feasibility of state monitoring, we tested whether N100 was preferentially enhanced when stimulation occurred in the S4 microstate across electrode densities (64/32/21/9 channels; ANOVA details in Supplementary Table 2). Across montages and conditions, TEPs showed a clear N100 within ~75–150 ms (Figure 4B–C,E,F,H,I,K,L). Descriptively, superimposed S4 versus random waveforms showed a larger (more negative) N100 in S4 in several montages (Figure 4C,F,I,L; mean ± SEM), but the corrected pairwise results were density‐specific. Significant pairwise effects and effect sizes are summarized in Table 1, with full post hoc results for each montage provided in Tables S3A–D.

**TABLE 1:** Significant pairwise contrasts in N100 amplitude and corresponding effect sizes across electrode densities.

Electrode montage	State pair	Mean(State A) (μV)	Mean(State B) (μV)	Δμ (μV)	*p*_adj_
64‐ch	S1–S2	−2.04	−1.17	−0.87	0.0125
64‐ch	S2–S4	−1.17	−2.38	1.21	0.0300
32‐ch	S1–S2	−2.10	−1.26	−0.84	0.0127
32‐ch	S2–S4	−1.26	−2.48	1.22	0.0337
21‐ch	S1–S2	−1.81	−0.93	−0.87	0.0245

*Note:* Electrode Montage, EEG electrode montage (64, 32, or 21 channel configuration). State pair, pair of microstates being compared (Mean(State A) – Mean(State B)). Mean(State A), Mean(State B), group‐mean N100 amplitudes for State A and State B, respectively (more negative values indicate larger N100 deflections). Δμ (μV), mean difference in N100 amplitude (Mean(State A) – Mean(State B)). *p*_adj_, *p* value after Bonferroni correction for multiple comparisons within each electrode density.

For the 64‐channel montage (Figure 4A–C), microstate significantly modulated N100 amplitude (*F* = 5.07, *p* = 0.0108; partial *η* ^2^ = 0.22). Post hoc tests within the frontal ROI showed S4 < S2 and S1 < S2 (more negative N100 for S4 and S1; Figure 4A). Relative to random, S4 produced a markedly larger N100 (Figure 4C). The 32‐channel montage replicated these findings (Figure 4D–F), with a significant microstate effect (*F* = 5.60, *p* = 0.007; partial *η* ^2^ = 0.24) and significant contrasts S4 versus S2 (*p* = 0.0337) and S1 versus S2 (*p* = 0.0127) (Figure 4D); S4 again showed the largest N100 among microstate‐defined conditions (Figure 4F).

With 21 channels (Figure 4G–I), the microstate main effect remained significant (*F* = 4.91, *p* = 0.0184; partial *η* ^2^ = 0.21), but only S1 versus S2 remained significant (*p* = 0.0245). S4‐related contrasts were no longer significant, showing only a weak trend for S4 versus S2 (*p* = 0.117; Figure 4H), although the ordering of mean N100 amplitudes was consistent with higher‐density montages. With 9 channels (Figure 4J–L), S4 still tended to yield a more negative N100, but no pairwise comparisons reached significance (Figure 4J).

To compare electrode densities directly, we derived state‐adjusted estimated marginal means (EMMs) from a linear mixed‐effects model including State as a fixed effect (Figure 4M). Bonferroni‐corrected comparisons showed no differences among the 64‐, 32‐, and 21‐channel montages (“ns”), whereas the 9‐channel montage differed significantly from both the 64‐ and 21‐channel configurations (*) (Figure 4N).

## 4 Discussion

This study proposed a whole‐brain, state‐guided TMS‐EEG strategy based on EEG microstates and showed that TMS pulses delivered during specific global brain states were associated with more stable and enhanced N100 responses. When stimulation was synchronized with time windows dominated by the S4 microstate, the clearest amplification effect was observed for the peak N100 TEP amplitude, whereas GMFA and LMFA did not reach statistical significance and should therefore be considered exploratory. The electrode‐density analysis showed that the S4‐related N100 enhancement was retained after downsampling from 64 to 32 channels, suggesting that this effect could be preserved under moderate spatial downsampling. However, no S4‐related pairwise contrast survived correction at 21 or 9 channels. Our findings indicated that this whole‐brain, state‐guided N100 enhancement protocol provided a stable and operationally reliable N100 readout.

Specifically, we investigated how pre‐stimulation whole‐brain patterns relate to the subsequent N100 and found that N100 was consistently largest when microstate S4 dominated the pre‐TMS interval. The clearest statistically supported effect was observed in the local TEP measure. GMFA and LMFA also showed the numerically largest N100 values under S4, but these differences did not reach statistical significance and should therefore be considered exploratory only. At the whole‐brain level, N100‐GMFA was numerically largest under S4, with only modest differences across the remaining conditions, but this pattern was not statistically significant. At the stimulation target (F3 electrode), S4 also produced the numerically largest N100‐LMFA, although this ROI‐level finding was descriptive only. Peak local TEP analyses confirmed that S4 yielded significantly greater N100 amplitude. These findings suggest that S4 may represent a candidate brain state associated primarily with larger local TEP N100 responses, whereas the GMFA and LMFA results showed numerical S4‐related trends that did not reach statistical significance and should therefore be interpreted as exploratory.

The study provided empirical evidence supporting a whole‐brain, state‐guided protocol to enhance N100 and extended the microstate‐locking framework proposed by Ding et al. [35]. While Ding et al. showed that microstates at stimulation systematically modulate TEP topography and N100 amplitude, our study demonstrated that EEG microstates can serve as a state variable for identifying pre‐stimulation brain states associated with larger N100 responses, in contrast to approaches relying on local oscillatory phase or power [63, 64]. Microstates reflected large‐scale, synchronized network activity on the order of tens to hundreds of milliseconds and provided an effective tool for characterizing millisecond‐scale whole‐brain dynamics [33, 34], aligning with evidence that late TEP components around 100 ms are primarily driven by distributed network dynamics rather than purely local activity [31]. Previous work has noted that approaches defining local brain states based on oscillatory frequency, amplitude, or phase were vulnerable to several limitations, including low signal‐to‐noise ratio, non‐sinusoidal waveform morphology, and rapid amplitude or frequency drift [65]. Taken together, using global microstates such as S4 to time stimulation offered two advantages: it better aligned with the large‐scale inhibitory networks indexed by the N100 component, and it reduced dependence on high signal‐to‐noise ratios.

In this study, the S4 state exhibited distinctive electrophysiological properties, which we interpreted as a “pre‐activated” configuration that facilitated specific large‐scale brain responses. Its scalp topography was centrally symmetric, with higher cumulative field strength over frontotemporal and central regions than other states; this polarity‐independent intensity suggests broader cortical engagement. Despite the highest field strength, S4 exhibited the lowest spatiotemporal complexity, as indexed by scalp complexity. Spatiotemporal Lempel–Ziv complexity (from binarized channel‐activation patterns) was minimal for S4, indicating a more restricted topographic repertoire and more regular temporal sequences. S4 therefore reflected a pre‐activated yet globally stable brain state: high‐amplitude activity in frontoparietal/central regions emerges in relatively stereotyped patterns rather than highly random ones. When TMS was delivered during S4, N100 amplitudes were maximal, indicating that external perturbations evoked larger responses when the brain was in this unified, high‐intensity, low‐complexity “pre‐activated” state.

Importantly, the S4‐related pattern was not abolished after accounting for pre‐stimulus global amplitude. In the covariate‐adjusted analysis, the overall State effect remained significant, and S4 showed the highest adjusted N100 amplitude. Post hoc contrasts showed that S4 exhibited significantly larger absolute N100 amplitudes than S2, S3, and S5 but did not differ significantly from S1. These findings suggest that the microstate‐guided approach may provide a methodological strategy for enhancing TMS‐evoked N100 responses. However, given the limited sample size, S4 should not be interpreted as a definitively optimal pre‐stimulation state. Instead, consistent with previous methodological work on state‐informed TMS‐EEG strategies [66], S4 may serve as a candidate state for N100 enhancement in future microstate‐guided TMS‐EEG studies.

We interpreted this finding in the context of the TMS‐evoked N100 component, which was not an isolated response of a single brain network but was widely regarded as an electrophysiological marker of cortico‐cortical interactions [67], and thought to be driven by cyclic large‐scale network dynamics. Our pre‐stimulation findings complement the poststimulation microstate analysis reported by Lucarelli and colleagues [36], suggesting that TMS‐evoked responses depend on both early stimulus‐driven dynamics and the brain state at stimulation onset. Accordingly, a pre‐activated S4 state, characterized by stronger initial distributed drive and greater cortical excitability, may provide favorable initial conditions for coupling TMS perturbations more efficiently into large‐scale circuits, thereby yielding larger and more reliable N100 responses. This aligned with the observation that TMS administered during the S4 state produced larger N100 amplitudes. Taken together, we proposed that the combination of high initial excitability and inter‐network stability in S4 may provide favorable conditions for efficient perturbation–network coupling, enabling robust state‐guided enhancement of N100. However, given that TEPs inevitably contain sensory and peripheral artifacts [68, 69], we conservatively interpreted the enhanced N100 in S4 as a larger scalp recorded response, rather than definitive evidence of heightened cortical excitability or superior perturbation network coupling. In the present study, the TMS‐evoked electrophysiological findings were derived from offline analyses rather than real‐time closed‐loop monitoring. Real‐time TMS‐EEG monitoring requires immediate neural readout during the experiment, as well as sophisticated technical approaches and dedicated software solutions [24, 70]. Although microstate classification was performed offline, it helped identify candidate whole‐brain states associated with larger evoked responses. Thus, retrospective microstate analysis might provide a useful step toward future real‐time state estimation and closed‐loop EEG‐guided TMS by offering candidate state templates or benchmarks [71]. Compared with existing EEG‐guided approaches that primarily relied on local oscillatory phase or power [47, 48], this framework characterized brain state at the whole‐brain topographic level and may better capture the distributed network dynamics underlying late TEP components such as the N100 [31].

Furthermore, we assessed the robustness of the S4‐related N100 enhancement with different EEG spatial sampling densities. After downsampling from 64 to 21 channels, state‐adjusted EMMs showed no differences in N100 amplitude among 64‐, 32‐, and 21‐channel montages, suggesting comparable overall N100 estimates across these configurations. However, the N100 amplitude results remained most consistent in the 64‐ and 32‐channel configurations. In the 21‐channel configuration, only the S1 versus S2 contrast survived correction and no S4‐related pairwise contrast remained significant, whereas in the 9‐channel configuration, the pairwise contrasts were lost. However, even with only nine channels, the relative ordering and detectability of N100 amplitudes in the S4 state were preserved, suggesting that low‐density montages may capture the broad qualitative features of the S4‐related pattern, but not its statistical specificity. These findings provided a methodological basis for the clinical use of low‐density EEG and supported the use of S4 as the preferred stimulation state for future N100‐based closed‐loop designs.

To address the limited stability of the TMS‐evoked N100 component, which was strongly influenced by trial‐to‐trial variability in activity within the target cortical region, we proposed a whole‐brain, state‐guided strategy for N100 enhancement. Our results demonstrated that delivering TMS pulses during specific pre‐activated microstates consistently increased N100 amplitude. In the electrode‐density analysis, the corrected S4‐related contrast was retained at 64 and 32 channels but not at 21 or 9 channels, indicating that S4‐related statistical specificity was preserved under moderate downsampling but not across all low‐density montages. These findings supported the potential feasibility of our approach for future attempts to stabilize and amplify N100 responses through stimulation targeted to specific microstate patterns. Accordingly, we outlined an application‐oriented, two‐stage pathway: (i) a state‐classification phase, in which high‐density EEG is used to characterize, at the population level, the distribution and features of pre‐activated microstates; and (ii) a triggering phase, in which simplified low‐density EEG is used to estimate whether the current state is a candidate state for enhanced N100 responses and, within a closed‐loop control framework, to trigger stimulation within the corresponding time window so as to stabilize and enhance the N100.

## 5 Limitations

This study has several limitations. First, the neural networks underlying the pre‐activated scalp state that facilitated N100 remain unclear. Second, electrode‐density effects were estimated by channel subsampling rather than true low‐density caps, potentially introducing sampling/layout bias; validation with dedicated low‐density montages is needed. Third, only healthy adults were studied, and the relatively older age profile of the present cohort may limit direct generalization to younger adult samples and clinical populations. Moreover, the present findings should be replicated in larger and more diverse independent cohorts to confirm the robustness and generalizability of the S4‐related N100 effect. Thus, while our findings validate the state sensitivity of the N100, translating this approach into therapeutic neuromodulation requires further validation through longitudinal studies in clinical populations. Finally, analyses were offline; future work will develop real‐time microstate‐triggered closed‐loop TMS‐EEG and test the robustness and clinical feasibility of state‐guided N100 modulation under clinically viable low‐density EEG.

## 6 Conclusion

This study proposed a whole‐brain, state‐guided TMS‐EEG strategy based on EEG microstates to stabilize and amplify the N100 component. We showed that stimulation delivered during the S4 microstate reliably produced the largest N100 responses, with the clearest effect observed in the TEP measure, whereas GMFA and LMFA were included as complementary exploratory measures. Electrode‐density analyses showed that the Bonferroni‐corrected S4 versus S2 contrast was retained at 64 and 32 channels, but no S4‐related pairwise contrast survived correction at 21 or 9 channels. Therefore, within this offline framework, the present study provides a microstate‐guided methodological approach for identifying candidate brain states associated with larger N100 responses, particularly when sufficient spatial sampling is available.

## Funding

This work was supported by the Brain Science and Brain‐like Intelligence Technology ‐National Science and Technology Major Project under Grant 2021ZD0204300.

## Ethics Statement

This study was approved by the ethics committee of Beijing Normal University (Approval number: CNL_A_0010_010).

## Conflicts of Interest

The authors declare no conflicts of interest.

## Supporting information


**Table S1A:** Covariate‐adjusted estimated marginal means.
**Table S1B:** S4‐focused post hoc contrasts.
**Table S2:** Repeated‐measures ANOVA of Microstate (S1–S5) on N100 amplitude across electrode densities.
**Table S3A:** Pairwise comparisons of N100 amplitude between microstates (64‐channel).
**Table S3B:** Pairwise comparisons of N100 amplitude between microstates (32‐channel).
**Table S3C:** Pairwise comparisons of N100 amplitude between microstates (21‐channel).
**Table S3D:** Pairwise comparisons of N100 amplitude between microstates (9‐channel).
**Figure S1:** N100 TEP amplitudes across cortical regions. (A) Schematic scalp map illustrating the five additional regions of interest (ROIs): right frontal, left parietal, right parietal, left occipital, and right occipital. (B–F) N100 peak amplitudes (mean ± SEM) for microstates S1–S5 and the Random condition in each ROI: (B) right frontal, (C) left parietal, (D) right parietal, (E) left occipital, and (F) right occipital. Bar plots showed that the right frontal and left parietal ROIs exhibited a clear S4‐related maximum, consistent with the state dependence observed in the primary DLPFC ROI and global GMFA, whereas N100 amplitudes in right parietal and occipital ROIs were smaller, more variable, and showed only modest microstate‐related differences.

## Data Availability

The datasets used and/or analyzed during the current study are available from the corresponding author on reasonable request.
