# Supplementary material for: State‐Guided TMS‐EEG for N100 Enhancement Study Based on Whole‐Brain EEG Microstates
Source: CNS Neurosci Ther. 2026 Jun 15;32(6):e70975. doi: 10.1002/cns.70975 (PMC13267666; doi:10.1002/cns.70975)
Supplement: Supplementary file 1 — Table S1A: Covariate‐adjusted estimated marginal means. Table S1B: S4‐focused post hoc contrasts. Table S2: Repeated‐measures ANOVA of Microstate (S1–S5) on N100 amplitude across electrode densities. Table S3A: Pairwise comparisons of N100 amplitude between microstates (64‐channel). Table S3B: Pairwise comparisons of N100 amplitude between microstates (32‐channel). Table S3C: Pairwise comparisons of N100 amplitude between microstates (21‐channel). Table S3D: Pairwise comparisons of N100 amplitude between microstates (9‐channel). Figure S1: N100 TEP amplitudes across cortical regions. (A) Schematic scalp map illustrating the five additional regions of interest (ROIs): right frontal, left parietal, right parietal, left occipital, and right occipital. (B–F) N100 peak amplitudes (mean ± SEM) for microstates S1–S5 and the Random condition in each ROI: (B) right frontal, (C) left parietal, (D) right parietal, (E) left occipital, and (F) right occipital. Bar plots showed that the right frontal and left parietal ROIs exhibited a clear S4‐related maximum, consistent with the state dependence observed in the primary DLPFC ROI and global GMFA, whereas N100 amplitudes in right parietal and occipital ROIs were smaller, more variable, and showed only modest microstate‐related differences. [file CNS-32-e70975-s001.docx]

**Supplementary**

**State-guided TMS-EEG for N100 Enhancement study based on Whole-Brain EEG Microstates**

Jiale Lan^1^, Yong Wang^2^, Xiaoli Li^1,3*^, He Chen^1*^

^1^ School of Automation Science and Engineering, South China University of Technology, Guangzhou, 510641, China

^2^ Department of Rehabilitation Medicine, Zhujiang Hospital, Southern Medical University, Guangzhou, 510000, China

^3^ Division of Psychology, Beijing Normal University, Beijing, China

* Corresponding author:

He Chen, E-mail address: chenhe@scut.edu.cn

Xiaoli Li, Email address: xiaolili@scut.edu.cn

**Covariate-adjusted analysis of the microstate effect on N100 amplitude**

To examine whether the observed microstate effect on N100 amplitude remained after accounting for differences in pre-TMS global amplitude, we performed a covariate-adjusted linear mixed-effects model. State was treated as a fixed effect, pre-TMS global amplitude was treated as a covariate, and subject was included as a random intercept. Supplementary Table 1 summarises the covariate-adjusted estimated marginal means for each microstate (1A) and the Bonferroni-corrected S4-focused post hoc contrasts (1B). These results showed that S4 exhibited the largest adjusted absolute N100 amplitude. However, the adjusted S4 versus S1 contrast was not significant, whereas S4 remained significantly larger than S2, S3, and S5.

Supplementary Table 1A. Covariate-adjusted estimated marginal means

| **State** | **Adjusted |N100|** | **SE** | **95% CI lower** | **95% CI upper** |
| --- | --- | --- | --- | --- |
| S1 | 2.005 | 0.275 | 1.467 | 2.544 |
| S2 | 1.153 | 0.273 | 0.619 | 1.687 |
| S3 | 1.633 | 0.273 | 1.097 | 2.168 |
| S4 | 2.452 | 0.283 | 1.897 | 3.006 |
| S5 | 1.629 | 0.272 | 1.095 | 2.163 |

Supplementary Table 1B. S4-focused post hoc contrasts

| **Contrast** | **Estimate** | **SE** | **df** | **t value** | $\boldsymbol{p}_{\mathbf{uncorr}}$ | $\boldsymbol{p}_{\boldsymbol{adj}}$ | **Significance** |
| --- | --- | --- | --- | --- | --- | --- | --- |
| S4 vs S1 | 0.446 | 0.300 | 89 | 1.49 | 0.140 | 0.562 | **ns** |
| S4 vs S2 | 1.299 | 0.292 | 89 | 4.44 | <0.001 | <0.001 | ******* |
| S4 vs S3 | 0.819 | 0.295 | 89 | 2.78 | 0.007 | 0.027 | ***** |
| S4 vs S5 | 0.823 | 0.290 | 89 | 2.83 | 0.006 | 0.023 | ***** |

***Estimate****, adjusted mean difference in N100 amplitude between S4 and each comparison state.*

***SE****, standard error.*

$\boldsymbol{p}_{\boldsymbol{uncorr}}$*, uncorrected p-value*

$\boldsymbol{p}_{\boldsymbol{adj}}$*, p value after Bonferroni correction across the four S4-focused contrasts.*

***95% CI lower*** ***and******95% CI upper****, lower and upper bounds of the 95% confidence interval for the adjusted mean, respectively.*

***ns****, not significant;* ****, p*** *< 0.05;* ******, p*** *< 0.001.*

**Main effect of Microstate on N100 amplitude**

We calculated the full repeated-measures analysis of variance (ANOVA) and post hoc test results for all electrode densities (64/32/21/9 channels).

Table 2 summarised the results of the repeated-measures ANOVA for the microstate factor State (S1–S5) on N100 amplitude at each electrode density, including F-statistics, uncorrected and Greenhouse–Geisser-corrected p-values, and partial η² as the effect size estimate. These results confirmed a significant main effect of State across all four electrode densities, with effect sizes ranging from moderate to large.

Supplementary Table 2. Repeated-measures ANOVA of Microstate (S1–S5) on N100 amplitude across electrode densities

| **Electrode Montage** | **F (4,72)** | $\boldsymbol{p}_{\mathbf{uncorr}}$ | $\boldsymbol{p}_{\boldsymbol{adj}}$ | **Partial η²** |
| --- | --- | --- | --- | --- |
| 64 -ch | 5.07 | 0.0012 | 0.0108 | 0.220 |
| 32-ch | 5.60 | 0.0006 | 0.0070 | 0.237 |
| 21-ch | 4.91 | 0.0015 | 0.0184 | 0.214 |
| 9-ch | 5.03 | 0.0012 | 0.0058 | 0.218 |

***F(4,72),*** *F statistic for the main effect of State;*

$\boldsymbol{p}_{\boldsymbol{uncorr}}$*, uncorrected p-value based on the standard F distribution assuming sphericity;*

$\boldsymbol{p}_{\boldsymbol{adj}}$*, p-value after Greenhouse–Geisser correction for potential violations of sphericity; Partial η², effect size for the State main effect, indicating the proportion of variance explained by State relative to the sum of State and error variance (η² > 0.14 reflects a large effect).*

**Post-hoc comparisons between microstates**

Supplementary Tables 3A–3D presented the complete set of pairwise post hoc comparisons of N100 amplitude between microstates (S1–S5) for all electrode montages (64, 32, 21, and 9 channels). For each contrast, the tables reported mean N100 amplitudes, mean differences (Δμ), and both uncorrected and Bonferroni-corrected p-values. In medium-to-high density configurations (64 and 32 channels), S1 and S4 consistently produced significantly larger negative N100 amplitudes than S2 ($\boldsymbol{p}_{\boldsymbol{adj}}$ < 0.05). Under the 21-channel configuration, the S1–S2 contrast remained significant, whereas the S2–S4 comparison no longer survived correction. For the 9-channel montage, several contrasts involving S4 (for example, S3–S4 and S2–S4) yielded uncorrected p-values below 0.01 but did not reach significance after Bonferroni correction, likely reflecting reduced statistical power under sparse spatial sampling. Taken together, these results indicated that state-guided N100 enhancement—characterised by larger deflections in S4 and S1 relative to S2—was most robust in high-density recordings; although the directional pattern was preserved at lower densities, statistical strength decreased as electrode density was reduced.

Supplementary Table 3A. Pairwise comparisons of N100 amplitude between microstates (64-channel)

| **Electrode Montage** | **State A** | **State B** | $\boldsymbol{Mean(State A)}$ **(µV)** | $\boldsymbol{Mean(State B)}$ **(µV)** | **Δµ (µV)** | $\boldsymbol{p}_{\boldsymbol{adj}}$ |
| --- | --- | --- | --- | --- | --- | --- |
| 64-ch | S1 | S2 | -2.04 | -1.17 | -0.87 | 0.0125 |
| 64-ch | S2 | S4 | -1.17 | -2.38 | 1.21 | 0.0300 |
| 64-ch | S2 | S3 | -1.17 | -1.65 | 0.48 | 0.1612 |
| 64-ch | S1 | S3 | -2.04 | -1.65 | -0.39 | 0.2752 |
| 64-ch | S3 | S4 | -1.65 | -2.38 | 0.73 | 0.3894 |
| 64-ch | S2 | S5 | -1.17 | -1.63 | 0.46 | 0.6283 |
| 64-ch | S1 | S5 | -2.04 | -1.63 | -0.41 | 0.7106 |
| 64-ch | S4 | S5 | -2.38 | -1.63 | -0.75 | 0.9881 |
| 64-ch | S1 | S4 | -2.04 | -2.38 | 0.34 | 1.0000 |
| 64-ch | S3 | S5 | -1.65 | -1.63 | -0.02 | 1.0000 |

$\boldsymbol{Mean(State A)}$***, $\boldsymbol{Mean(State B)}$,*** *group-mean N100 amplitudes for State A and* *State B****,*** *respectively (more negative values indicate larger N100 deflections).****Δµ (µV),*** *mean difference in N100 amplitude ($Mean(State A)$ − $Mean(State B)$.
$\boldsymbol{p}_{\boldsymbol{adj}}$, p value after Bonferroni correction for multiple comparisons within each electrode density.*

Supplementary Table 3B. Pairwise comparisons of N100 amplitude between microstates (32-channel)

| **Electrode Montage** | **State A** | **State B** | $\boldsymbol{Mean}\mathbf{(}\boldsymbol{State} \boldsymbol{A}\mathbf{)}$ **(µV)** | $\boldsymbol{Mean}\mathbf{(}\boldsymbol{State} \boldsymbol{B}\mathbf{)}$ **(µV)** | **Δµ (µV)** | $\boldsymbol{p}_{\boldsymbol{adj}}$ |
| --- | --- | --- | --- | --- | --- | --- |
| 32-ch | S1 | S2 | -2.10 | -1.26 | -0.84 | 0.0127 |
| 32-ch | S2 | S4 | -1.26 | -2.48 | 1.22 | 0.0337 |
| 32-ch | S2 | S3 | -1.26 | -1.69 | 0.43 | 0.1427 |
| 32-ch | S1 | S3 | -2.10 | -1.69 | -0.41 | 0.1593 |
| 32-ch | S3 | S4 | -1.69 | -2.48 | 0.79 | 0.2351 |
| 32-ch | S1 | S5 | -2.10 | -1.61 | -0.49 | 0.3466 |
| 32-ch | S4 | S5 | -2.48 | -1.61 | -0.87 | 0.5049 |
| 32-ch | S1 | S4 | -2.10 | -2.48 | 0.38 | 1.0000 |
| 32-ch | S2 | S5 | -1.26 | -1.61 | 0.35 | 1.0000 |
| 32-ch | S3 | S5 | -1.69 | -1.61 | -0.08 | 1.0000 |

Supplementary Table 3C. Pairwise comparisons of N100 amplitude between microstates (21-channel)

| **Electrode Montage** | **State A** | **State B** | $\boldsymbol{Mean(State A)}$ **(µV)** | $\boldsymbol{Mean(State B)}$ **(µV)** | **Δµ (µV)** | $\boldsymbol{p}_{\boldsymbol{adj}}$ |
| --- | --- | --- | --- | --- | --- | --- |
| 21-ch | S1 | S2 | -1.81 | -0.93 | -0.87 | 0.0245 |
| 21-ch | S2 | S4 | -0.93 | -2.51 | 1.58 | 0.1172 |
| 21-ch | S1 | S3 | -1.81 | -1.39 | -0.42 | 0.2495 |
| 21-ch | S2 | S3 | -0.93 | -1.39 | 0.45 | 0.2898 |
| 21-ch | S3 | S4 | -1.39 | -2.51 | 1.12 | 0.3094 |
| 21-ch | S1 | S5 | -1.81 | -1.27 | -0.54 | 0.4115 |
| 21-ch | S4 | S5 | -2.51 | -1.27 | -1.24 | 0.5029 |
| 21-ch | S1 | S4 | -1.81 | -2.51 | 0.70 | 1.0000 |
| 21-ch | S2 | S5 | -0.93 | -1.27 | 0.33 | 1.0000 |
| 21-ch | S3 | S5 | -1.39 | -1.27 | -0.12 | 1.0000 |

Supplementary Table 3D. Pairwise comparisons of N100 amplitude between microstates (9-channel)

| **Electrode Montage** | **State A** | **State B** | $\boldsymbol{Mean(State A)}$ **(µV)** | $\boldsymbol{Mean(State B)}$ **(µV)** | **Δµ (µV)** | $\boldsymbol{p}_{\boldsymbol{adj}}$ |
| --- | --- | --- | --- | --- | --- | --- |
| 9-ch | S3 | S4 | -2.14 | -3.29 | 1.15 | 0.0573 |
| 9-ch | S2 | S4 | -1.79 | -3.29 | 1.49 | 0.0607 |
| 9-ch | S1 | S5 | -2.68 | -1.92 | -0.76 | 0.1731 |
| 9-ch | S4 | S5 | -3.29 | -1.92 | -1.36 | 0.1734 |
| 9-ch | S1 | S2 | -2.68 | -1.79 | -0.89 | 0.3274 |
| 9-ch | S1 | S3 | -2.68 | -2.14 | -0.54 | 0.4950 |
| 9-ch | S1 | S4 | -2.68 | -3.29 | 0.61 | 1.0000 |
| 9-ch | S2 | S3 | -1.79 | -2.14 | 0.35 | 1.0000 |
| 9-ch | S2 | S5 | -1.79 | -1.92 | 0.13 | 1.0000 |
| 9-ch | S3 | S5 | -2.14 | -1.92 | -0.22 | 1.0000 |

**State-guided N100 across brain regions**

To further examine the spatial specificity of the state-guided N100 enhancement beyond the primary DLPFC ROI and global measures, we conducted an exploratory analysis of N100 peak amplitudes in five additional regions of interest (ROIs): right frontal, left parietal, right parietal, left occipital, and right occipital. The right frontal ROI comprised electrodes Fp2, F2, F4, AF4, FC2, AF8, F6 , FC6 and FC4; the left parietal ROI comprised P7, P5, P3, P1, CP3, CP5, TP7 and CP1; the right parietal ROI comprised P8, P6, P4, P2, CP4, CP6, TP8 and CP2; the left occipital ROI comprised O1, PO7, and PO3; and the right occipital ROI comprised O2, PO8, and PO4. For each ROI, N100 peak amplitudes were extracted for microstates S1–S5 and the Random condition and are presented as bar plots (mean ± SEM).


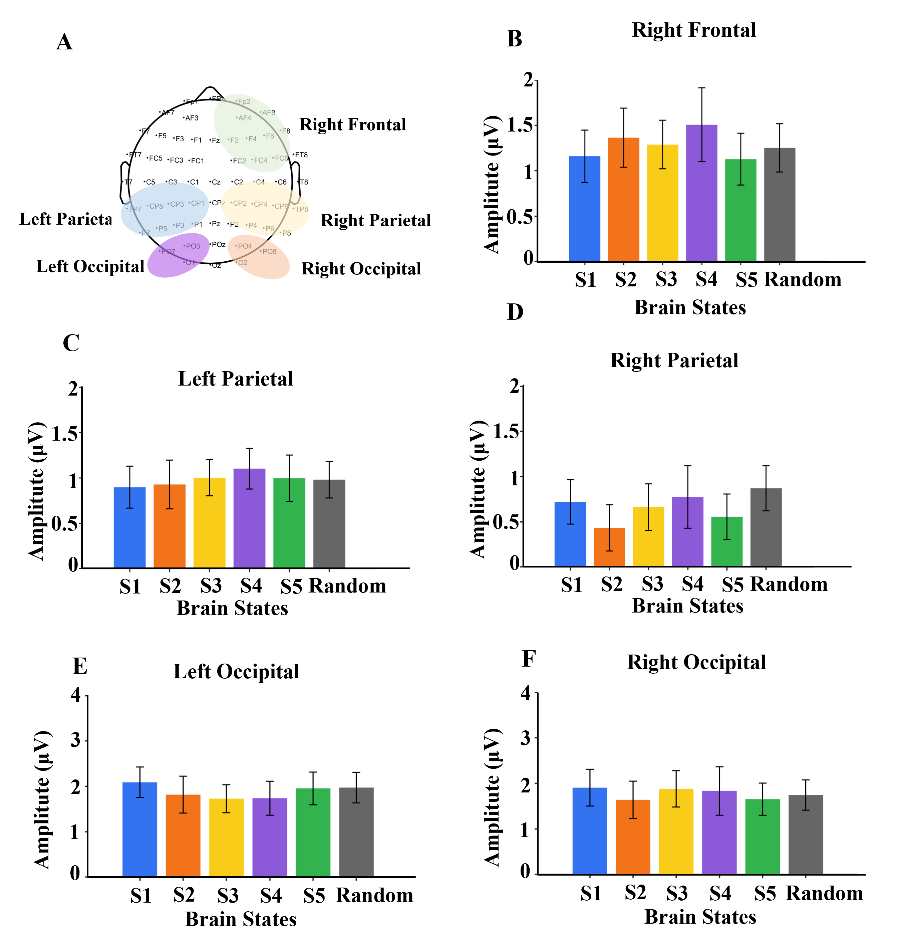


Supplementary Figure S1. N100 TEP amplitudes across cortical regions. (A) Schematic scalp map illustrating the five additional regions of interest (ROIs): right frontal, left parietal, right parietal, left occipital, and right occipital. (B–F) N100 peak amplitudes (mean ± SEM) for microstates S1–S5 and the Random condition in each ROI: (B) right frontal, (C) left parietal, (D) right parietal, (E) left occipital, and (F) right occipital. Bar plots showed that the right frontal and left parietal ROIs exhibited a clear S4-related maximum, consistent with the state dependence observed in the primary DLPFC ROI and global GMFA, whereas N100 amplitudes in right parietal and occipital ROIs were smaller, more variable, and showed only modest microstate-related differences.

As shown in Supplementary Figure S1, the right frontal and left parietal ROIs exhibited a clear S4-related pattern, with S4 tending to produce the most negative N100 amplitudes. This closely mirrored the state dependence observed in the primary DLPFC ROI and in the global GMFA measure. By contrast, N100 amplitudes in the right parietal and bilateral occipital ROIs were smaller and more variable overall, and state-related differences in these regions were modest. These exploratory results complemented the main GMFA, LMFA, and local TEP findings. They indicated that the pre-activated S4 state preferentially amplified the N100 within a fronto-parietal network functionally coupled to the stimulated DLPFC.
